# Very-Low-Density Lipoproteins Quantity but Not Composition Is Altered in Normotriglyceridemic Subjects with Elevated Lipoprotein (a) Level

**DOI:** 10.3390/ijms27020556

**Published:** 2026-01-06

**Authors:** Ewa Wieczorek-Breitzke, Martyna Feliksiak, Agnieszka Kuchta, Maciej Jankowski, Agnieszka Ćwiklińska

**Affiliations:** 1 Department of Clinical Chemistry, Faculty of Pharmacy, Medical University of Gdańsk, 80-211 Gdansk, Poland; agnieszka.kuchta@gumed.edu.pl (A.K.); maciej.jankowski@gumed.edu.pl (M.J.); agnieszka.cwiklinska@gumed.edu.pl (A.Ć.); 2 Central Clinical Laboratory, University Clinical Centre in Gdańsk, 80-952 Gdansk, Poland; mfeliksiak@uck.gda.pl

**Keywords:** apolipoproteins, lipoprotein (a), normotriglyceridemia, triglycerides, very-low-density lipoproteins

## Abstract

Cardiovascular disease (CVD) is influenced by disturbances in lipoprotein composition and metabolism, including triglyceride-rich lipoproteins (TRLs) and elevated lipoprotein (a) (Lp(a)). While interactions between Lp(a) and very-low-density lipoproteins (VLDL) have been studied in hypertriglyceridemic and CVD populations, data in normotriglyceridemic individuals without CV events are limited. Seventy normotriglyceridemic adults with triglycerides < 150 mg/dL and no CV events were enrolled and divided into two groups based on Lp(a) concentration: <30 mg/dL and ≥30 mg/dL. VLDL was isolated by ultracentrifugation, and concentrations of Lp(a), lipids (triglycerides, cholesterol), and apolipoproteins (apo B, apo C-II, apo C-III, apo E) were measured in serum and VLDL. Serum lipid and apolipoprotein concentrations did not differ between the groups. Individuals with Lp(a) ≥ 30 mg/dL had significantly higher VLDL concentrations of triglycerides (+71%), cholesterol (+54%), apo B (+28%), apo C-II (+36%), and apo C-III (+33%). Ratios of lipids and apolipoproteins to apo B indicated unchanged VLDL particle composition, suggesting that differences reflected increased particle number rather than altered composition. In normotriglyceridemic subjects with Lp(a) ≥ 30 mg/dL, VLDL particles are more abundant but compositionally unchanged. Redistribution of lipids and apolipoproteins toward VLDL may contribute to VLDL residual cardiovascular risk, underscoring the need for further studies on VLDL-Lp(a) interactions.

## 1. Introduction

Cardiovascular disease (CVD) remains major global health concern, responsible for nearly 30% of all deaths worldwide despite significant advances in prevention and treatment strategies [1]. Among the principal contributors to atherosclerotic cardiovascular disease (ASCVD) development and resulting cardiovascular (CV) events are disruptions in lipid metabolism and lipoprotein composition. Elevated levels of low-density lipoprotein (LDL) cholesterol (LDL-C) are recognized as the primary lipid-related risk factor for CVD [2]. However, disturbances in the metabolism of other lipoprotein classes, including triglyceride-rich lipoproteins (TRL), such as very-low-density lipoproteins (VLDL), resulting in changes in VLDL composition and/or the development of hypertriglyceridemia (HTG), also play a crucial role in the pathogenesis of CVD [3].

HTG, defined as a serum triglyceride (TG) concentration above 150 mg/dL, is a well-established risk factor for CVD [4]. Epidemiological and clinical evidence supports a causal link between elevated serum TG levels, the number of TRL and their remnants, and an increased risk of developing atherosclerosis, CVD, and CV events (myocardial infarction, stroke, unstable angina, acute coronary syndrome, sudden cardiac arrest, or heart failure). However, recent studies increasingly indicate that not only HTG resulting from an increased TG concentration in TRL but also elevated number of TRL particles and alterations in their composition—occurring even in the absence of HTG—may contribute to the development of atherosclerosis and CVD [3].

Another atherogenic lipoprotein is lipoprotein (a) (Lp(a)), whose concentration in the blood is approximately 90% genetically determined [5]. The Lp(a) particle is highly heterogeneous due to the presence of various isoforms of its protein component—apolipoprotein(a) (apo(a)). The type of isoform is of considerable importance, as the presence of smaller apo(a) isoforms (characterized by several repeats of the kringle IV type 2 domain) is associated with significantly higher plasma Lp(a) concentrations compared to larger isoforms (30–40 kringle IV type 2 domain repeats) [6]. Lp(a) exhibits both pro-inflammatory properties (including activation of granulocytes and monocytes, increase levels of pro-inflammatory cytokines, and stimulation of smooth muscle cell proliferation and migration) and prothrombotic effects (such as reduced fibrinolytic activity and thrombus stabilization), which contribute to the initiation and progression of atherosclerosis and CVD. Elevated Lp(a) levels ≥ 30 mg/dL are observed in approximately 10–30% of the population [7] and have been identified as an independent cardiovascular risk factor [5,8]. Importantly, both changes in VLDL composition and increased concentration of Lp(a) may increase the likelihood of cardiovascular events even when LDL-C levels are within the recommended range, being residual cardiovascular risk factors [3,8].

Most previous studies investigating the relationship between Lp(a) and TG have focused on populations with mixed hyperlipidemia, CVD, or CV events, where HTG is a common component. These studies have reported an inverse correlation between Lp(a) and TG concentrations in both serum and VLDL [9]. Furthermore, a greater number and larger size of VLDL particles have been associated with lower Lp(a) levels [9,10].

The aim of this study was to assess the VLDL composition in normotriglyceridemic individuals with elevated Lp(a) level but no CV events or other comorbidities that could affect VLDL metabolism/composition.

## 2. Results

### 2.1. Lipid and Apolipoprotein Concentrations in Serum

The study group (Lp(a) ≥ 30 mg/dL) did not differ from the control group (Lp(a) < 30 mg/dL) in terms of serum TG, total cholesterol (TC), LDL-C, LDL-C adjusted for Lp(a), small dense LDL-C (sdLDL-C), non-high-density lipoprotein cholesterol (non-HDL-C), high-density lipoprotein cholesterol (HDL-C), phospholipids, and apolipoproteins (apo) levels (Table 1). After adjusted for multiple comparisons using the Benjamini–Hochberg false discovery rate (BH-FDR) method, the results remained unchanged (Lp(a) q < 0.001, effect size: r = 2.31; TG q = 0.276, r = 0.19; TC q = 0.412, d = 0.12; LDL-C _Friedewald_ q = 0.398, d = 0.15; HDL-C q = 0.521, r = −0.08; apo A-I q = 0.487, r = −0.10, apo B q = 0.331, r = 0.17).

### 2.2. Lipid and Apolipoprotein Composition of VLDL

**Figure 1:**
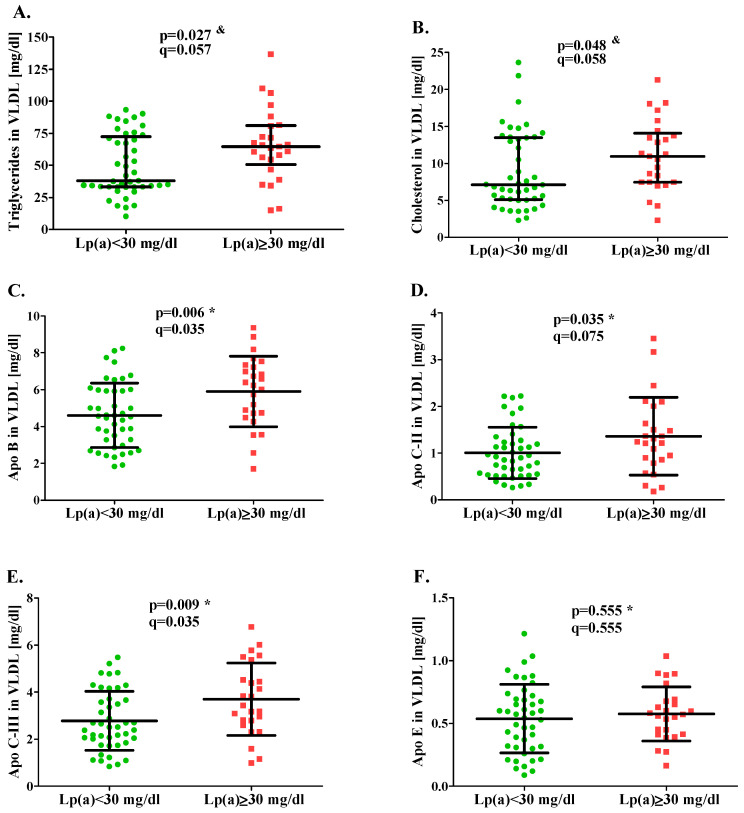
VLDL concentration of triglycerides (**A**), cholesterol (**B**), apolipoprotein B (**C**), apolipoprotein C-II (**D**), apolipoprotein C-III (**E**), and apolipoprotein E (**F**) in the group with Lp(a) < 30 mg/dL (green circles) and Lp(a) ≥ 30 mg/dL (red squares). Data are presented as mean ± standard deviation (SD) or median (25th and 75th percentiles); the normality of the distribution of results was assessed using the Shapiro–Wilk test; * unpaired Student’s *t*-test (parametric distribution); ^&^ the Mann–Whitney test (non-parametric distribution); *p* < 0.05 considered as statistically significant. Symbols “*p*” indicate nominal statistical significance (*p* < 0.05), whereas symbols “q” indicate significance after adjustment for multiple comparisons using the Benjamini–Hochberg false discovery rate (BH-FDR) method. Abbreviations: VLDL—very-low-density lipoproteins, Lp(a)—lipoprotein (a), apo—apolipoprotein.

The median TG concentration in VLDL for the study group was 65 mg/dL, and it was higher compared to the control group by 71% on average (*p* = 0.027; Figure 1); however, this difference reached only borderline significance after the BH-FDR correction and was associated with a small-to-moderate effect size (r = 0.27). Median VLDL cholesterol (CH) level was also significantly higher in the group with Lp(a) ≥ 30 mg/dL compared to the group with Lp(a) < 30 mg/dL (by 54% on average; *p* = 0.048), showing borderline significance after BH-FDR adjustment and a small effect size (r = 0.24).

In contrast, the mean concentration of VLDL-associated apolipoproteins, apo B, apo C-II, and apo C-III were 5.90 mg/dL, 1.36 mg/dL, and 3.70 mg/dL, respectively, and were significantly higher in the study group compared with the control group (by 28%, 36%, and 33% on average). After BH-FDR correction, differences in VLDL apo B (*p* = 0.006; q = 0.035) and apo C-III (*p* = 0.009; q = 0.035) remained statistically significant, with moderate effect sizes (Cohen’s d = 0.71 and 0.63, respectively), whereas the difference in apo C-II did not remain significant after correction (q = 0.075). No significant difference in VLDL apo E concentration was observed between the groups (*p* = 0.555).

Lipid and apolipoprotein concentrations in VLDL were converted to apo B in VLDL, which allowed for the assessment of the composition of VLDL particles. The ratios of TG, CH, apo C-II, apo C-III, and apo E to apo B particle in VLDL did not differ between the study and control groups. The results are presented in Table 2. Consistent with unadjusted analyses, no differences in VLDL particle composition were observed after the BH-FDR correction.

To further explore global multivariate patterns in VLDL particle composition, principal component analysis (PCA) was performed. The first principal component explained 78.7% of the total variance, whereas the second component explained 8.8%, indicating that most variability in VLDL composition was captured by a single dominant component. The PCA score plot (PC1 vs. PC2) did not reveal distinct clustering or separation of individuals according to Lp(a) status, suggesting the absence of major multivariate differences in VLDL particle composition.

The relative content of lipids and apolipoproteins in VLDL in relation to their total serum concentration was also assessed (Table 3). It was observed that the average relative content of TG in VLDL was significantly higher in individuals with Lp(a) ≥ 30 mg/dL compared to those with Lp(a) < 30 mg/dL (*p* = 0.038). There were no significant differences in the relative content of CH in VLDL (*p* = 0.106). The percentage of apo B in VLDL relative to the total serum pool of this apolipoprotein was significantly higher in subjects with Lp(a) ≥ 30 mg/dL compared to those with Lp(a) < 30 mg/dL. Similarly, the relative content of exchangeable apolipoproteins in VLDL—apo C-II and apo C-III—was also higher in the study group. The relative content of apo E in VLDL did not differ statistically significantly between the groups (*p* = 0.077) (Table 3). After the BH-FDR correction, the contribution of VLDL apo B, apo C-II, and apo C-III to the serum pool remained significantly higher in the high Lp(a) group, whereas the contribution of VLDL triglycerides showed only a trend toward higher values.

## 3. Discussion

Disorders in the metabolism of TRL play a significant role in the development of CVD and resulting CV events [3]. The adverse impact of TRL disturbances, including VLDL, may result from an increase in the number of VLDL particles or a change in their lipid–protein composition, which affects their atherogenicity [3,9]. Another important, independent, and genetically determined (approximately 90%) risk factor for CVD is Lp(a), of which elevated concentrations ≥ 30 mg/dL are observed in approximately 10–30% of the population [7]. Importantly, both disturbances in VLDLs as well as elevated Lp(a) levels increase the risk of CV events, even in subjects with target LDL-C concentrations, representing the significant components of residual cardiovascular risk [11].

In our study, we found that in normolipidemic subjects with Lp(a) ≥ 30 mg/dL, the number of VLDL particles increased without changing their composition.

Most previous studies on changes in VLDL composition and the link between VLDL and Lp(a) have been conducted in study groups characterized by a wide spectrum of lipid disorders, including severe HTG, as well as the presence of comorbidities such as diabetes or obesity [9,10,12]. Additionally, documented experiences from other researchers focusing on VLDL changes have typically examined them in patients with specific pathological conditions, such as coronary artery disease [13]. There are also reports on genetic disorders, for example, mutations in the *Proprotein convertase subtilisin/kexin 9* (*PCSK9*) gene, in connection with the study of lipoprotein metabolism kinetics [14]. Studies have also been conducted on postprandial metabolic changes [15]. However, there is a lack of data on the composition and quantity of VLDLs in individuals without HTG, whose risk factor is elevated Lp(a) but who have not experienced CV events. Our study did not aim to investigate the effects of HTG or other disease states but rather to assess changes in the composition or quantity of VLDL in individuals without HTG and CV events, in whom the variable was an elevated Lp(a) concentration.

Interestingly, our observation regarding TG in VLDL in normotriglyceridemic subjects differs from the results observed by others that were performed in subjects with lipid disturbances and/or CVD and that showed an inverse relationship between Lp(a) and VLDL-TG concentration. Werba et al., Ramos-Cáceres et al., and Marco-Benedí et al. demonstrated an inverse correlation between Lp(a) and TG in serum and in VLDL, which became particularly evident at high TG concentrations (above 300 mg/dL) [9,10,12]. These results suggest that increased hepatic synthesis of VLDL in the context of HTG may suppress Lp(a) production [9]. Furthermore, a greater number and larger size of VLDL particles have been associated with lower Lp(a) levels [9,10]. This relationship may be driven by enhanced VLDL production and competition between VLDL remnants and Lp(a) for hepatic LDL receptors (LDLR) because binding to LDLR is one of the possible pathways of Lp(a) cellular uptake and elimination from the blood [16,17,18]. This indicates that TRL metabolism may influence Lp(a) concentration.

Since the study groups in our research did not exhibit HTG, documented CV events, or chronic diseases that could affect TG metabolism, our results show that the relationship between VLDL and Lp(a) may change in individuals with HTG compared to those with normotriglyceridemia, which may be due to a different metabolism of VLDL in individuals with HTG (increased synthesis and/or decreased catabolism).

Regarding the analysis of apolipoprotein composition in VLDL, higher levels of apo B, apo C-II, and apo C-III were observed in the group with Lp(a) above 30 mg/dL; importantly, these differences remained statistically significant after BH-FDR correction and were associated with small-to-moderate effect sizes. Apo C-II is an activator of lipoprotein lipase (LPL), the enzyme responsible for plasma TRL catabolism. Higher apo C-II levels in VLDL could suggest potentially more efficient metabolism of these lipoproteins. On the other hand, apo C-III is an LPL inhibitor, and its elevated concentration promotes HTG development [11,19]. However, the results presented in Table 2 and Table 3 showed that the increase in apo C-II and apo C-III concentration in VLDL is due to an increased number of VLDL particles rather than changes in VLDL composition affecting their catabolic efficiency. The apo C-II-to-apo C-III ratio in VLDL, indicating the effectiveness of VLDL catabolism in plasma, did not differ between the study and control groups (0.36 ± 0.15 vs. 0.36 ± 0.11, *p* = 0.918.

To characterize the composition of VLDL particles, lipid and apolipoprotein concentrations in VLDL were converted to apo B concentration in VLDL, considering that each VLDL particle contains one apo B molecule [20]. We found that VLDL particles did not differ in composition between the groups. Notably, the absence of differences in VLDL particle composition was consistent across unadjusted analyses and after false discovery rate (FDR) correction and was accompanied by small effect sizes. These results indicate that the observed differences in absolute concentrations of VLDL components between the groups were due to an increased number of VLDL particles in individuals with Lp(a) ≥ 30 mg/dL rather than a change in VLDL composition. This may be caused by increased hepatic production of VLDL in individuals with elevated Lp(a) while maintaining VLDL effective plasma catabolism [16]. VLDL serves, in a sense, a substrate necessary for Lp(a) synthesis, as LDL particles—a component of Lp(a)—are formed through VLDL catabolism.

Analysis of the apo E concentration in VLDL and the apo E/apo B ratio in VLDL revealed no statistically significant differences between the study and the control group. The lack of difference in apo E concentration in VLDL between the groups differs from the results obtained by others [10,14]. Ramos-Cáceres demonstrated that a significant inverse relationship and higher apo E levels in VLDL were associated with lower Lp(a) concentrations. Moreover, they found that subjects carrying the apoE2/E2 genotype had significantly lower levels of Lp(a) [10]. On the other hand, Croyal et al. found a strong positive correlation between apo E in VLDL and Lp(a). Their study showed that the more apo E-rich VLDL particles were synthesized, the more Lp(a) was produced [14]. The authors concluded that apo E content in VLDL may be a key factor influencing Lp(a) synthesis. When analyzing the reasons for this discrepancy, the characteristics of the study population should be considered, as Ramos-Cáceres et al. examined a group of individuals with HTG [10], whereas the positive correlation observed by Croyal et al. was found in a study on a very specific group of subjects with PCSK9 or LDLR gene mutations [14]. The absence of a correlation between apo E in VLDL and Lp(a) in our study and various relationships observed by others for different patient populations can suggest the link between apo E and Lp(a) that can be related/affected by disturbances in lipoprotein metabolism. Apo E is a multifunctional protein with a very important role in lipid metabolism, and many studies have shown the association between apo E polymorphism and disturbances in the pathogenesis of numerous diseases, including atherosclerosis and obesity [21]. The main antiatherogenic apo E role is to mediate the clearance of atherogenic lipoproteins from plasma circulation via its interaction with LDL receptor family proteins and heparan sulfate proteoglycans. Thus, its optimal expression is crucial for normal metabolism of TG-rich lipoproteins. However, it has been shown that apo E overexpression and/or accumulation may contribute to HTG by stimulating VLDL-TG production and by impairing VLDL lipolysis [22]. Moriarty et al. concluded that differences in affinity of apo E for lipoprotein clearance receptors may affect Lp(a) catabolism and suggested a competition between Lp(a) and apo E for similar receptors [23]. So, it seems that the link between VLDL-apo E and Lp(a) may concern both lipoprotein synthesis [14] and clearance [23], but the exact mechanisms are not known and further studies are needed.

In this study, we further assessed the proportional content of lipids and apolipoproteins in VLDL relative to their total serum concentrations. We found that the percentage content of TG, apo B, apo C-II, and apo C-III in VLDL was significantly higher in the group with Lp(a) ≥ 30 mg/dL levels compared to the control group. The observed increase in the relative content of TG and apolipoproteins in VLDL in the study group, despite the absence of differences in their serum concentrations, suggests a shift in these components toward the VLDL fraction. This indicates an elevation in their concentration within VLDL in the group with Lp(a) ≥ 30 mg/dL, which—given the lack of differences when lipids and apolipoproteins were converted to apo B in VLDL—further implies an increased number of VLDL particles in individuals with elevated Lp(a) levels.

However, the percentage differences between the groups for individual lipid and apolipoprotein contents differed, which can be related with VLDL heterogeneity. VLDL particles are very heterogeneous in size and composition, and different VLDL subclasses can be distinguished, for instance, larger TG-enriched VLDL particles (VLDL-1) and smaller VLDL-2 particles containing less TG [24]. VLDL subclasses can also differ in the contents of exchangeable apolipoproteins, such as apo Cs and apo E, that affect its metabolism [25]. Different percentage differences between the Lp(a) < 30 mg/dL and Lp(a) ≥ 30 mg/dL groups for individual lipids and apolipoproteins in VLDL can be related with differences in the concentration of individual VLDL subfractions. This issue needs further study.

It is important to acknowledge the limitations of our study. One of them is the relatively small number of participants, especially in the study group with elevated Lp(a) above 30 mg/dL (*n* = 25), which could influence statistical power. Sensitivity analysis indicated that the study was powered to reliably detect medium-to-large effects (MDE ≈ Cohen’s d 0.71 at α = 0.05 and 80% power). Therefore, the lack of between-group differences in VLDL compositional indices should be interpreted as an absence of large effects rather than definitive evidence of no effect, while the statistically significant results were generally associated with small-to-moderate effect sizes. Nevertheless, smaller compositional differences cannot be excluded and should be addressed in larger cohorts. Furthermore, several factors, including statin use, BMI, sex, and smoking status, may influence VLDL metabolism and represent potential confounders. Due to the limited sample size, fully adjusted multivariable regression models were not performed to avoid overfitting. However, the absence of global group separation in PCA and the small effect sizes observed across VLDL compositional parameters suggest that major compositional differences are unlikely to be driven by these factors. It is also worth noting that this study has a cross-sectional design, which does not allow us to determine the temporal sequence or establish a definitive causal relationship between VLDL and Lp(a). The study is based on concentration measurements, which are less advanced than kinetic studies (assessing production and catabolism rates), which could provide much more information about the dynamics of lipoprotein metabolism and the link between VLDL and Lp(a) in subjects without HTG. Additionally, besides measuring apo B concentration in VLDL that allows for direct estimation of lipoprotein particles number [24], it would be valuable to use other methods that allow for the direct determination of VLDL particle number and size such as NMR spectroscopy or electron microscopy. It is also worth noting that the apo B assay measures both apoB-100 (the structural protein of VLDL) and apoB-48 (the structural protein of chylomicrons and their remnants). However, taking into account the fact that apoB-48-containing lipoproteins typically represent <1% of the total concentration of circulating apo B-containing lipoproteins, even in a postprandial state, apoB-48-containing remnants was considered negligible, particularly given that all participants were fasting. Therefore, differences within isolated lipoproteins were considered only as differences in VLDL composition. It should also be mentioned that the size of Lp(a) particles is an important factor in research on these lipoproteins. Since Lp(a) size is genetically determined, a limitation of this study is the lack of the assessment of *LPA* gene polymorphism, which determines the apo(a) isoform size and accounts for over 90% of the variability in Lp(a) concentration in the blood [26]. Despite these limitations, our research indicates the need for further, more in-depth studies on the relationship between VLDL and Lp(a) in both normotriglyceridemic and hypertriglyceridemic subjects, as this relationship may be of a completely different nature depending on the level of TG.

## 4. Materials and Methods

### 4.1. Research Groups

Seventy adult subjects with no history of CV events at the time of recruitment and with TG serum concentration < 150 mg/dL, being under the care of the General Practitioner (Non-public Health-Care Center, Pomerania region, Poland), were recruited into the study. The exclusion criteria in the study were medical conditions that affect lipid and lipoproteins metabolism such as liver diseases, kidney diseases, diabetes, cancer, acute disease within 3 months before the study, and the use of fibrates, PCSK9 inhibitors, hormone replacement therapy, steroids, and immunosuppressive agents. Statin use was permitted, and the study groups were matched based on the preparation administered (Table 4). All participants gave written voluntary informed consent for participation in the study. The study was approved by the Bioethics Commission for Research of the Medical University of Gdańsk, Poland (No. KB/566/2023, KB/566-495/2024, KB/556-44/2025), and all procedures were conducted in accordance with the Declaration of Helsinki.

The individuals were allocated into two groups according to Lp(a) levels, namely, Lp(a) < 30mg/dL group (control group) and Lp(a) ≥ 30 mg/dL group (study group). The threshold value of 30 mg/dL was adopted to differentiate the two groups, as it has been demonstrated that CVD risk increases significantly when Lp(a) concentration exceeds 30 mg/dL [27]. The characteristics of the study groups are presented in Table 4.

The participants in the groups did not differ in terms of age, gender, statin usage frequency, smoking frequency, body mass, height, body mass index (BMI), and creatinine and glucose concentration, as well as aspartate aminotransferase (AST), alanine aminotransferase (ALT), and alkaline phosphatase (ALP) activity (Table 4).

### 4.2. Isolation of Serum and VLDL

**Figure 2:**
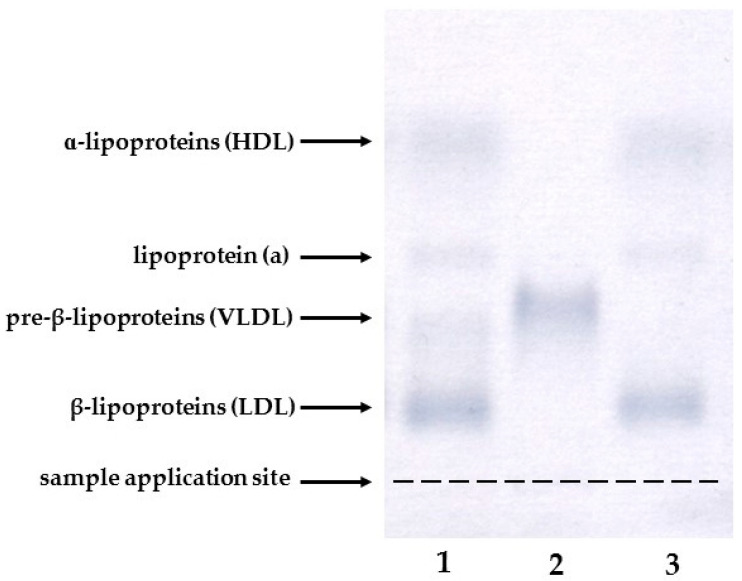
Representative electrophoresis image of serum (1), the isolated VLDL fraction (2), and the infranatant (3) containing the remaining lipoproteins (HDL, Lp(a), and LDL).

Venous blood samples were obtained from participants following an overnight fast and collected into commercially available serum-separating tubes (BD Vacutainer, Franklin Lakes, NJ, USA). Serum was isolated within one hour of collection by centrifuging whole blood at 2000× *g* for 10 min at room temperature. The VLDL were isolated via serum ultracentrifugation, as previously described [28]. In brief, 1.8 mL of serum was transferred into a 3.2 mL ultracentrifuge tube (Polyallomer Bell-top, Beckman Coulter, Brea, CA, USA) and carefully layered with NaCl solution (density = 1.006 g/mL). Ultracentrifugation was carried out using a Beckman Optima™ TLX Ultracentrifuge (Beckman Coulter, Brea, CA, USA) equipped with a fixed-angle rotor (TLA 100.3, Beckman Coulter, Brea, CA, USA) at 541,000× *g* for 60 min at 4 °C (acceleration 0, deceleration 9). Following ultracentrifugation, approximately 1 mL of the VLDL fraction was obtained after slicing the tube (Beckman Tube Slicer; Beckman Coulter, Brea, CA, USA). The purity of the VLDL fraction obtained after ultracentrifugation was confirmed by agarose gel electrophoresis (Hydragel 7 LIPO + Lp(a), Sebia, Lisses, France) (Figure 2).

### 4.3. Biochemical Analyses and Calculations

Serum and VLDL concentrations of TG, TC, and phospholipids were quantified using commercially available enzymatic assay kits from Wiener Lab (Rosario, Argentina) for TG and TC and Wako Diagnostics (Lexington, MA, USA) for phospholipids. Intra- and inter-assay coefficients of variation (CV) for the lipid and apolipoprotein measurements are presented in Table 5. The concentration of Lp(a) was determined spectrophotometrically using an assay kit from Randox Laboratories Polska Sp. z o.o. (Warsaw, Poland) and expressed in units [mg/dL]. The method used minimized the impact of apo(a) isoform heterogeneity on the Lp(a) determination result. For Lp(a) concentrations below the lower limit of the calibration curve (6.8 mg/dL), also adopted as the limit of quantification (LOQ), the Lp(a) concentration was assumed as value of LOQ/2 = 3.4 mg/dL [29]. Creatinine and glucose concentrations as well as ALT, AST, and ALP activity were measured using Wiener Lab (Rosario, Argentina) reagents. Non-HDL-C concentration was calculated by subtracting HDL-C from TC. The concentration of LDL-C was estimated using the Friedewald and Sampson-NIH equations. LDL-C levels calculated using the Friedewald equation also were adjusted for Lp(a) concentration using the Dahlen correction method, which assumes that the cholesterol content of Lp(a) accounts for approximately 30% of total Lp(a) mass [30]. The concentration of sdLDL was estimated using the Sampson equation, which enables the approximation of these atherogenic lipoproteins [31]. Apo A-I, apo B, apo C-II, apo C-III, and apo E concentrations were measured via immunonephelometry with reagent kits sourced from Siemens Healthcare (Erlangen, Germany) for apo A-I, apo B, and apo E and from Randox Laboratories Polska Sp. z o.o. (Warsaw, Poland) for apo C-II and apo C-III.

### 4.4. Statistical Analysis

Statistical analysis was performed using the STATISTICA 13 software (StatSoft Polska Sp. z o.o., Krakow, Poland) and GraphPad Prism 5 software (GraphPad Software, LLC, Boston, MA, USA). The normality of the distribution of results was assessed using the Shapiro–Wilk test. The categorical data were presented as numbers and percentages, and the differences between the groups were evaluated using the chi-square test. The continuous data were expressed as mean ± standard deviation (SD) for variables with a parametric distribution or as median with 25th and 75th percentiles for variables with a non-parametric distribution. Statistical differences between the study groups were evaluated using Student’s unpaired *t*-test or the Mann–Whitney test for parametric and non-parametric distributions, respectively. Statistical significance was considered at *p* < 0.05. To account for multiple testing, the Benjamini–Hochberg false discovery rate (BH-FDR) correction was applied separately within each family of analyses (Table 1, Table 2 and Table 3, and Figure 1). Both unadjusted *p*-values and FDR-adjusted *q*-values are reported. In addition to *p*-values, effect sizes were calculated to quantify the magnitude of between-group differences. Cohen’s d was used for parametric comparisons, r (Z/√N) for non-parametric comparisons, and Cramér’s V for categorical variables. Effect sizes were interpreted using conventional thresholds (Cohen’s d: 0.2 small, 0.5 medium, 0.8 large; r and Cramér’s V: 0.1 small, 0.3 medium, 0.5 large). Given the exploratory design and observed sample sizes, a sensitivity analysis (minimum detectable effect; MDE) was performed to estimate the effect size that could be reliably detected for two-sided comparisons, assuming α = 0.05 and 80% power. For *n* = 45 vs. *n* = 25, the estimated MDE corresponded to approximately Cohen’s d ≈ 0.71. Principal component analysis (PCA) was performed on standardized VLDL compositional variables using the correlation matrix to explore global multivariate patterns.

## 5. Conclusions

In individuals with Lp(a) ≥ 30 mg/dL but without HTG and CV events, higher concentrations of TG, CH, apo B, apo C-II, and apo C-III in VLDL were identified, despite no differences in total serum lipid or apolipoprotein levels. Converted VLDL lipids and apolipoproteins to apo B indicated that these differences reflect an increased number of VLDL particles rather than altered particle compositions. An increase in the amount of lipids and apolipoproteins in the VLDL fraction may contribute to increasing the residual cardiovascular risk related to TRL. In the light of these results, further, more extensive research examining the relationship between VLDL and Lp(a), as well as the composition of VLDL in non-HTG groups, appears especially relevant. Elucidating the link between Lp(a) and TRL metabolism—and its potential impact on cardiovascular risk—may be an important step toward designing targeted therapies that more effectively regulate levels of atherogenic lipoproteins. This may, in the future, contribute to a reduction in cardiovascular risk and enhance the effectiveness of CVD prevention strategies.

## Figures and Tables

**Table 1:** Lipid and apolipoprotein concentrations in serum.

Parameter	Control Group: Lp(a) < 30 mg/dL	Study Group: Lp(a) ≥ 30 mg/dL	*p*-Value
Lipoprotein (a) [mg/dL]	10 (3.4–19)	59 (40–75)	<0.001 ^&^
Triglycerides [mg/dL]	80 (56–109)	99 (83–115)	0.090 ^&^
Total cholesterol [mg/dL]	185 ± 36	189 ± 41	0.704 *
LDL-C _Friedewald_ [mg/dL]	113 ± 33	119 ± 32	0.455 *
LDL-C _Sampson-NIH_ [mg/dL]	114 ± 33	120 ± 32	0.431 *
LDL-C _Friedewald_ adjusted for Lp(a) [mg/dL]	109 ± 32	99 ± 35	0.234 *
sdLDL-C _calculated_ [mg/dL]	29 ± 9	33 ± 8	0.061 *
non-HDL-C [mg/dL]	129 ± 33	138 ± 31	0.285 *
HDL-C [mg/dL]	54 (46–64)	47 (43–58)	0.170 ^&^
Phospholipids [mg/dL]	248 ± 33	235 ± 27	0.230 *
Apo A-I [mg/dL]	197 (176–224)	205 (176–226)	0.945 ^&^
Apo B [mg/dL]	91 (83–108)	100 (88–105)	0.473 ^&^
Apo C-II [mg/dL]	4.21 ± 1.05	3.91 ± 1.26	0.298 *
Apo C-III [mg/dL]	11.30 (8.58–13.52)	11.26 (9.43–12.58)	0.851 ^&^
Apo E [mg/dL]	3.85 ± 1.01	3.41 ± 0.78	0.064 *

Data are presented as mean ± standard deviation (SD) or median (25th and 75th percentiles); the normality of the distribution of results was assessed using the Shapiro–Wilk test; * unpaired Student’s *t*-test (parametric distribution); ^&^ the Mann–Whitney test (non-parametric distribution); *p* < 0.05 considered as statistically significant. Abbreviations: LDL-C _Friedewald_—low-density lipoprotein cholesterol calculated using the Friedewald equation, LDL-C _Sampson-NIH_—low-density cholesterol calculated using the Sampson-NIH equataion, LDL-C _Friedewald_ adjusted for Lp(a)—low-density lipoprotein cholesterol calculated using the Dahlen correction, taking into account the Lp(a) cholesterol concentration, sdLDL-C—small dense low-density lipoprotein cholesterol, non-HDL-C—non-high-density lipoprotein cholesterol, HDL-C—high-density lipoprotein cholesterol, apo—apolipoprotein.

**Table 2:** The ratios of lipids and apolipoproteins in VLDL to apo B concentration in VLDL.

Parameter	Control Group: Lp(a) < 30 mg/dL	Study Group: Lp(a) ≥ 30 mg/dL	*p*-Value	q (BH-FDR)	Effect Size
TG_VLDL_/Apo B_VLDL_	9.87 (8.19–13.35)	10.46 (9.06–12.01)	0.596 ^&^	0.298	r = 0.15
CH_VLDL_/Apo B_VLDL_	1.85 ± 0.52	1.83 ± 0.37	0.825 *	0.825	d = −0.04
Apo C-II_VLDL_/Apo B_VLDL_	0.21 ± 0.07	0.22 ± 0.09	0.708 *	0.356	d = 0.20
Apo C-III_VLDL_/Apo B_VLDL_	0.61 ± 0.17	0.62 ± 0.14	0.709 *	0.424	d = 0.18
Apo E_VLDL_/Apo B_VLDL_	0.11 ± 0.04	0.10 ± 0.02	0.092 *	0.298	d = −0.43

Data are presented as mean ± standard deviation (SD) or median (25th and 75th percentiles); the normality of the distribution of results was assessed using the Shapiro–Wilk test; * unpaired Student’s *t*-test (parametric distribution); ^&^ the Mann–Whitney test (non-parametric distribution); *p* < 0.05 considered as statistically significant; *p*-values were adjusted for multiple comparisons using the Benjamini–Hochberg false discovery rate (BH-FDR) method. Effect sizes are reported as Cohen’s d for parametric comparisons and as r (calculated as Z/√N) for non-parametric comparisons. Abbreviations: TG—triglycerides, CH—cholesterol, VLDL—very-low-density lipoproteins, apo—apolipoprotein. These findings should be interpreted in the context of study power: the absence of significant differences in compositional indices is consistent with small effect sizes, indicating that large compositional changes are unlikely, although small effects cannot be excluded.

**Table 3:** Relative (%) content of lipids and apolipoproteins in VLDL in relation to their total serum concentration.

Parameter	Control Group: Lp(a) < 30 mg/dL	Study Group: Lp(a) ≥ 30 mg/dL	*p*-Value	q (BH-FDR)	Effect Size
TG _VLDL_ [%]	58 ± 14	65 ± 16	0.038 *	0.076	d = 0.53
CH _VLDL_ [%]	4.6 (2.7–7.4)	4.9 (3.7–7.7)	0.106 ^&^	0.159	r = 0.24
Apo B _VLDL_ [%]	5.0 (3.2–6.0)	6.7 (4.3–8.6)	0.017 ^&^	0.035	r = 0.29
Apo C-II _VLDL_ [%]	24.2 (15.6–29.3)	31.2 (26.1–37.0)	0.006 ^&^	0.035	r = 0.33
Apo C-III _VLDL_ [%]	25.7 ± 10.1	32.2 ± 10.7	0.014 *	0.035	d = 0.63
Apo E _VLDL_ [%]	14.0 (8.8–18.8)	17.6 (12.8–21.9)	0.077 ^&^	0.092	r = 0.22

Data are presented as mean ± standard deviation (SD) or median (25th and 75th percentiles); the normality of the distribution of results was assessed using the Shapiro–Wilk test; * unpaired Student’s *t*-test (parametric distribution); ^&^ the Mann–Whitney test (non-parametric distribution); *p* < 0.05 considered as statistically significant; *p*-values were adjusted for multiple comparisons using the Benjamini–Hochberg false discovery rate (BH-FDR) method. Effect sizes are reported as Cohen’s d for parametric comparisons and as r (calculated as Z/√N) for non-parametric comparisons. Abbreviations: TG—triglycerides, CH—cholesterol, apo—apolipoprotein.

**Table 4:** The characteristics of the research groups.

Parameter	All Participants	Control Group: Lp(a) < 30 mg/dL	Study Group: Lp(a) ≥ 30 mg/dL	*p*-Value
Number	70	45	25	--
Gender (female/male)	38/32 (54%/46%)	22/23 (49%/51%)	16/9 (64%/36%)	0.223 ^#^
Age [years]	41 ± 12	41 ± 12	43 ± 13	0.422 *
Body mass [kg]	79 ± 14	79 ± 13	80 ± 15	0.769 *
Height [cm]	172 ± 9	173 ± 10	172 ± 9	0.584 *
BMI [kg/m^2^]	27 ± 4	26 ± 4	27 ± 5	0.438 *
Rosuvastatin/Atorvastatin	10/3 (14%/4%)	5/2 (11%/4%)	5/1 (20%/4%)	0.612 ^#^
Smoking	5 (7%)	4 (9%)	1 (4%)	0.554 ^#^
Creatinine [mg/dL]	0.88 (0.73–1.03)	0.97 (0.78–1.03)	0.78 (0.72–0.93)	0.126 ^&^
Glucose [mg/dL]	91 (80–99)	92 (84–99)	82 (77–98)	0.130 ^&^
AST [U/L]	21 ± 6	22 ± 7	21 ± 6	0.614 *
ALT [U/L]	17 (14–26)	18 (13–28)	17 (14–22)	0.689 ^&^
ALP [U/L]	144 (111–171)	148 (122–176)	132 (94–161)	0.103 ^&^

Data are presented as mean ± SD or median (25th and 75th percentiles) or as the number and percentage of participants; the normality of the distribution of results was assessed using the Shapiro–Wilk test; ^#^ chi-square test; * unpaired Student’s *t*-test (parametric distribution); ^&^ the Mann-Whitney test (non-parametric distribution); *p* < 0.05 considered as statistically significant. Abbreviations: BMI—body mass index, AST—aspartate aminotransferase, ALT—alanine aminotransferase, ALP—alkaline phosphatase.

**Table 5:** Intra- and inter-assay coefficients of variation (CV) for the lipid and apolipoprotein measurements.

Parameter	Intra-Assay CV	Inter-Assay CV
Total cholesterol	1.8%	2.7%
Triglycerides	2.5%	7.7%
Phospholipids	1.7%	3.1%
Apolipoprotein A-I	3.1%	4.5%
Apolipoprotein B	3.8%	4.3%
Apolipoprotein C-II	6.4%	7.5%
Apolipoprotein C-III	7.5%	13.0%
Apolipoprotein E	4.1%	6.4%

## Data Availability

The original contributions presented in this study are included in the article. Further inquiries can be directed to the corresponding author.
